# Supplementary material for: Enhancement of activation-induced T cell proliferation by SIRPG in a CD47-independent manner
Source: Front Immunol. 2025 Nov 21;16:1668361. doi: 10.3389/fimmu.2025.1668361 (PMC12678352; doi:10.3389/fimmu.2025.1668361)
Supplement: Supplementary file 1 [file DataSheet1.pdf]

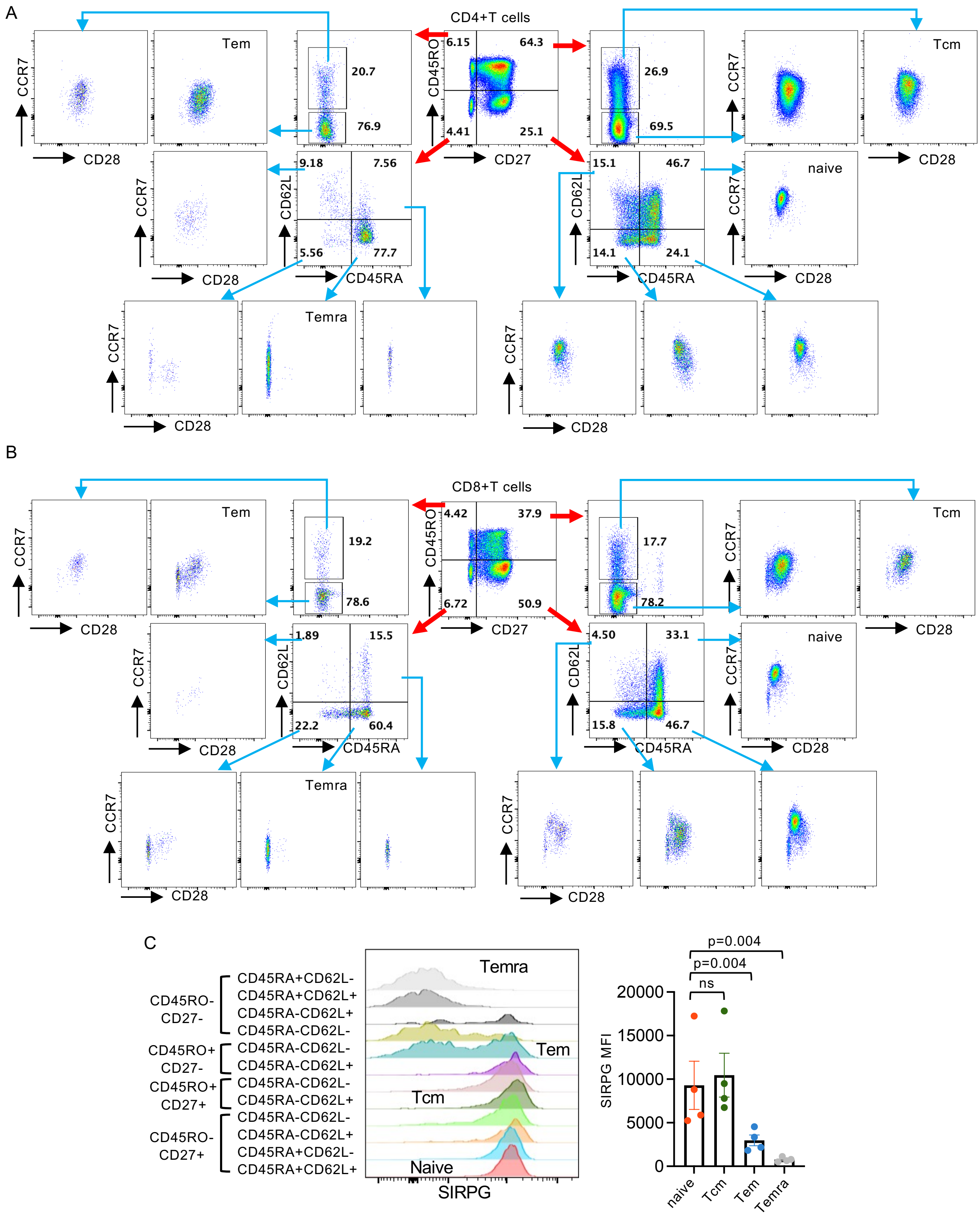

A
CD4+T cells
CD45RO
CD27
6.15
64.3
4.41
25.1
CD62L
CD45RA
9.18
7.56
5.56
77.7
15.1
46.7
14.1
24.1
20.7
76.9
26.9
69.5
CCR7
CD28
Tem
Tcm
naive
Temra
B
CD8+T cells
4.42
37.9
6.72
50.9
1.89
15.5
22.2
60.4
4.50
33.1
15.8
46.7
19.2
78.6
17.7
78.2
C
CD45RO-
CD27-
CD45RO+
CD27-
CD45RO+
CD27+
CD45RO-
CD27+
CD45RA+CD62L-
CD45RA+CD62L+
CD45RA-CD62L+
CD45RA-CD62L-
CD45RA-CD62L-
CD45RA-CD62L+
CD45RA-CD62L-
CD45RA-CD62L+
CD45RA-CD62L-
CD45RA-CD62L+
CD45RA+CD62L-
CD45RA+CD62L+
Temra
Tem
Tcm
Naive
SIRPG
SIRPG MFI
p=0.004
p=0.004
ns
20000
15000
10000
5000
0
naive
Tcm
Tem
Temra

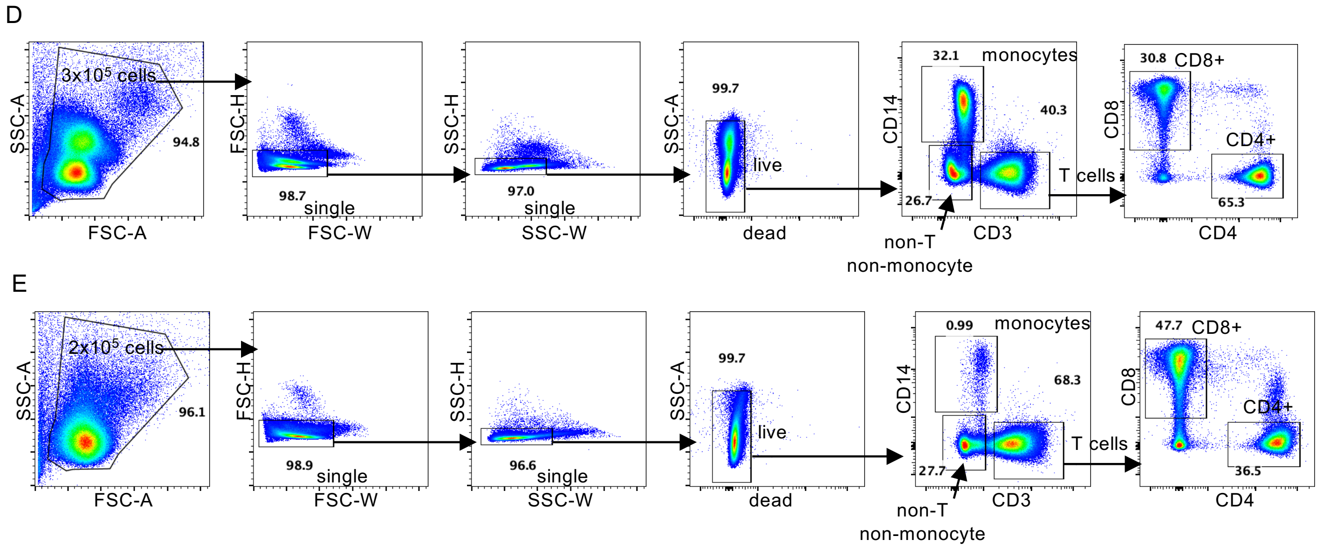


**Figure S1. The expression of SIRPG in peripheral blood and synovial T cells.** Peripheral blood CD4+ T cells (**A**), and CD8+ T cells (**B** & **C**) were stained with antibodies against indicated markers. The gating of various subsets of is shown in **A** and **B**. The overlay histograms of SIRPG of various subsets of CD8+ T cells are shown in **C**. Cumulative SIRPG MFI of indicated populations is shown in the bar graph of **C**. **D & E.** Gating strategy for identifying peripheral blood (**D**) and synovial fluid (**E**) CD8+ T cells is shown.

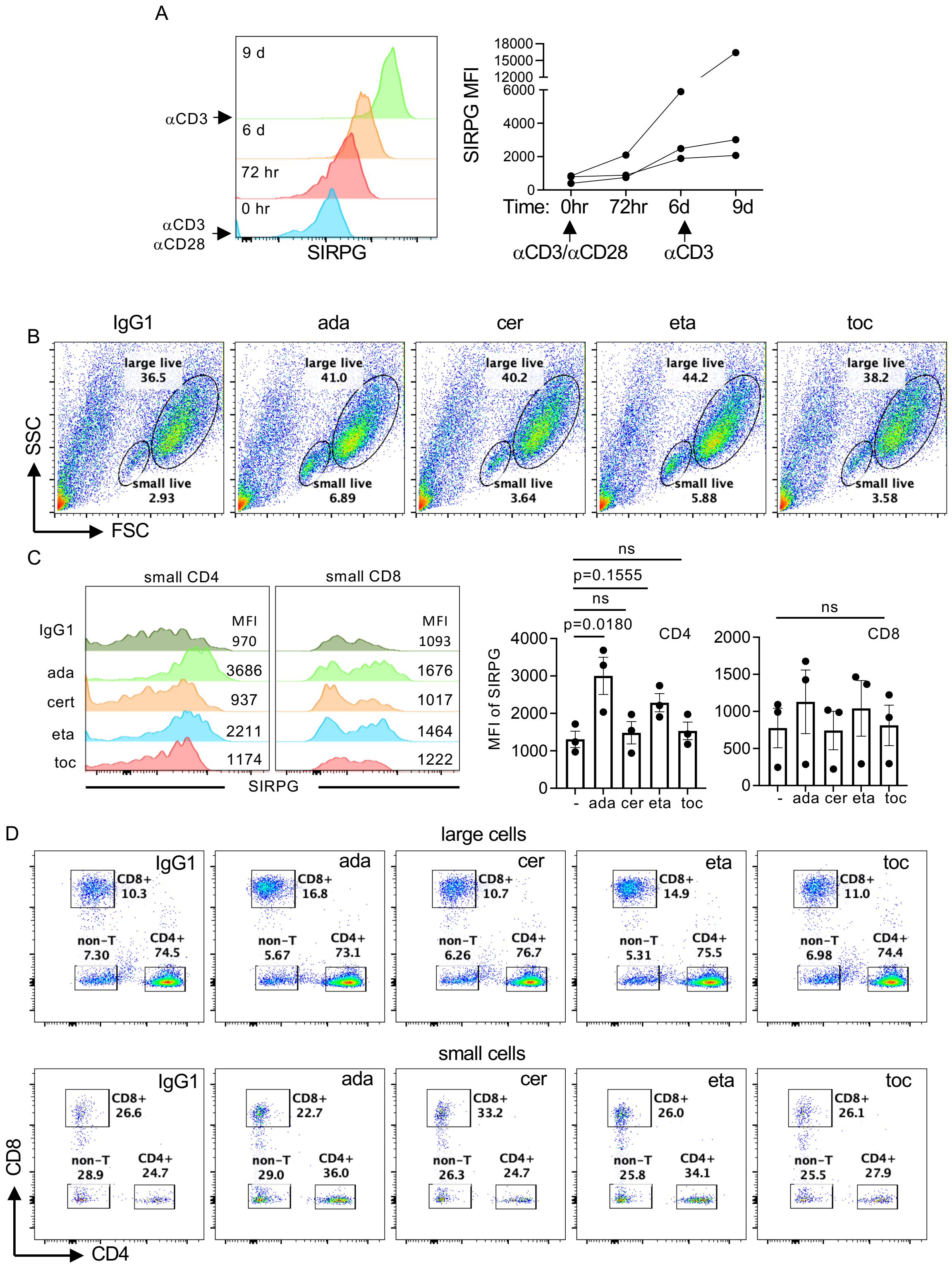


**Figure S2. Differential effects of TNFα inhibitors on the expression of SIRPG.** PBMC were stimulated with anti-CD3/anti-CD28 on day 0 and anti-CD3 on day 6 (A) or day 3 (B-D). The expression of SIRPG in CD8+ T cells was examined with FACS. Representative SIRPG overlaid histograms and cumulative SIRPG MFI are shown in the left and right panel of **A**, respectively. **B-D**. Ada, cert, eta, toc, or control IgG1 was added on day 0. The cells were analyzed with FACS to identify blasting (large live) and non-blasting cells (small live) (**B**). Representative overlaid SIRPG histograms and cumulative SIRPG MFI of non-blasting cells are shown in the left panel and right panel of **C**, respectively. Representative CD4/CD8 dot plots of blasting and non-blasting cells are shown in **D**.

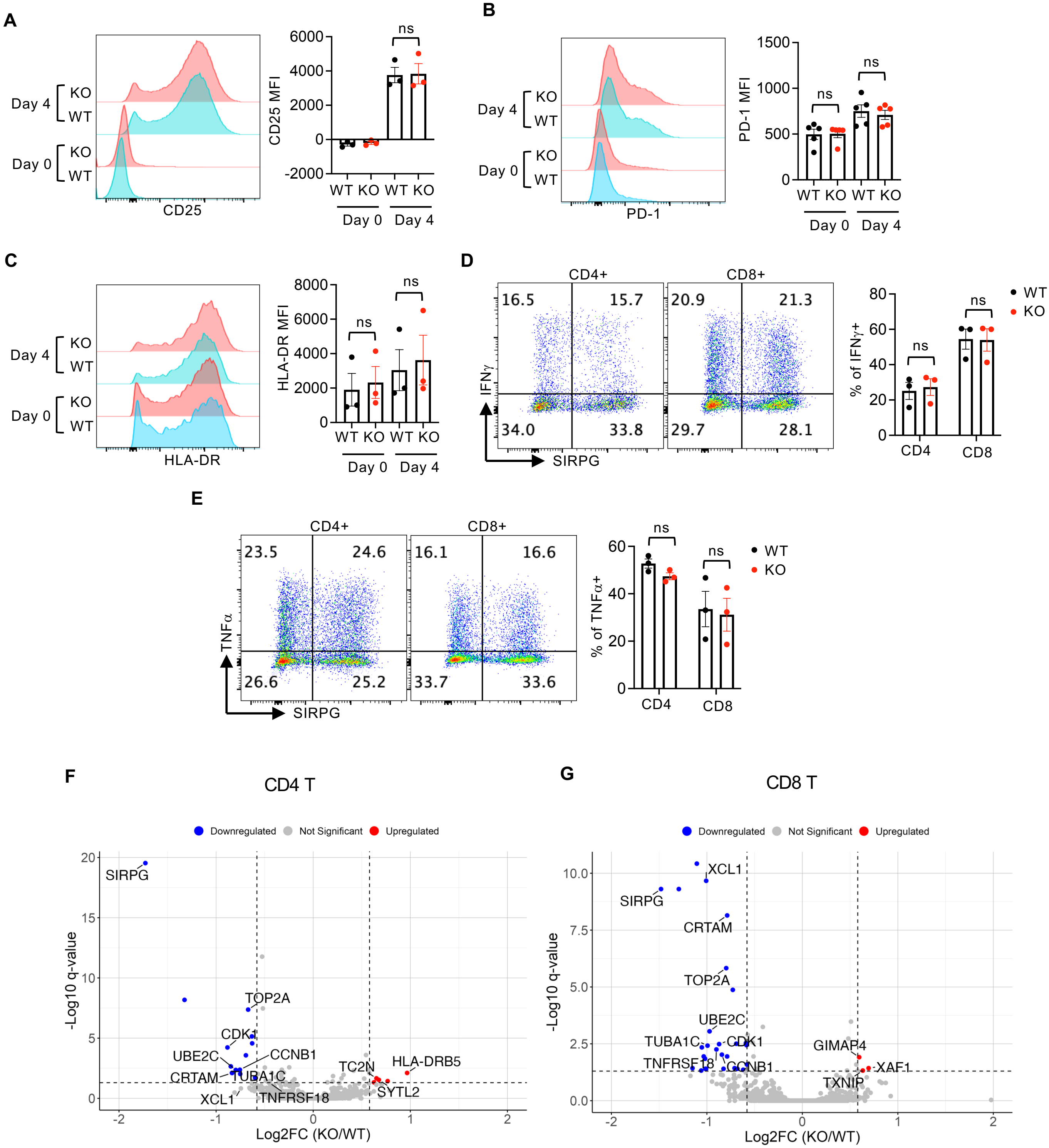
A
Day 4
KO
WT
Day 0
KO
WT
CD25
CD25 MFI
ns
WT KO WT KO
Day 0 Day 4
B
PD-1
PD-1 MFI
C
HLA-DR
HLA-DR MFI
D
CD4+
CD8+
16.5
15.7
34.0
33.8
20.9
21.3
29.7
28.1
IFNγ
SIRPG
% of IFNγ+
CD4
CD8
E
23.5
24.6
26.6
25.2
16.1
16.6
33.7
33.6
TNFα
% of TNFα+
F
CD4 T
Downregulated
Not Significant
Upregulated
-Log10 q-value
Log2FC (KO/WT)
SIRPG
TOP2A
CDK1
UBE2C
CCNB1
CRTAM
TUBA1C
XCL1
TNFRSF18
TC2N
HLA-DRB5
SYTL2
G
CD8 T
XCL1
SIRPG
CRTAM
TOP2A
UBE2C
TUBA1C
CDK1
TNFRSF18
CCNB1
GIMAP4
XAF1
TXNIP

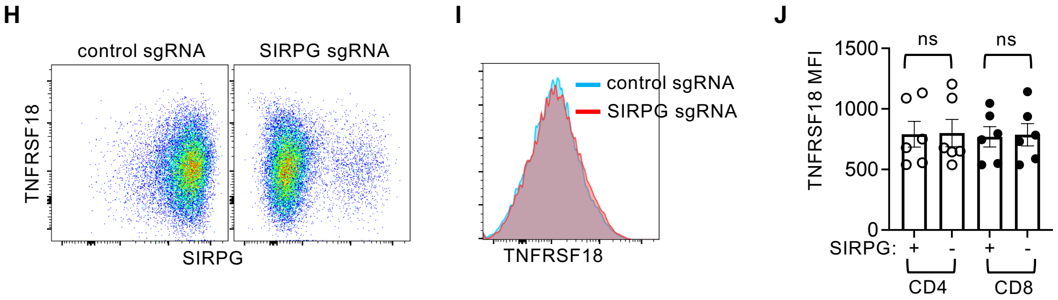


**Figure S3. Identification of SIRPG-regulated genes. A-C.** Control (WT) or SIRPG sgRNA (KO) transfected PBMC were stimulated with anti-CD3 for 4 days. The upregulation of CD25 (**A**), PD-1 (**B**), and HLA-DR (**C**) was examined with FACS. Representative overlaid histograms and cumulative MFI are shown. **D** & **E**. The WT and SIRPG KO cells were also mixed at 1:1 ratio, stimulated with anti-CD3, and subjected to intracellular staining for IFN$\gamma$ (**D**) and TNF$\alpha$ (**E**). Representative FACS plots and cumulative percentage of cytokine positive cells is shown. **F** & **G**. The differentially expressed genes between SIRPG+ and SIRPG- CD4+ T cells (**F**) and between SIRPG+ and SIRPG- CD8+ T cells (**G**) are shown in the volcano plots. **H-J**. The expression of TNFRSF18 of SIRPG+ and SIRPG- cells was examined with FACS. Representative SIRPG/TNFRSF18 dot plots are shown in **H**; representative TNFRSF18 overlay histograms are shown in **I**; and the TNFRSF18 MFI from all donors is shown in **J**.

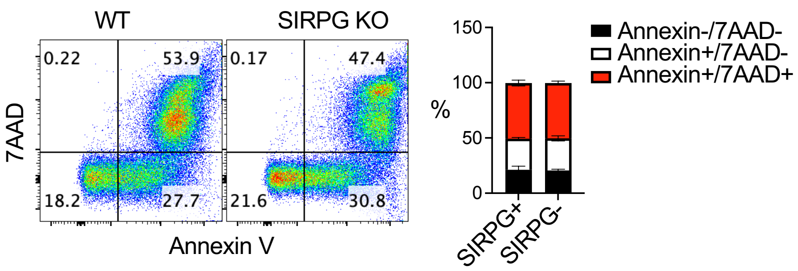


**Figure S4. Little impact of SIRPG on the apoptosis of T cells.** Stimulated WT and SIRPG KO T cells shown in Figure 4D were stained for the level of AnnexinV and 7AAD. Representative dot plots are shown in the left panel and the the percentage of indicated populations from three donors is shown in the right panel.

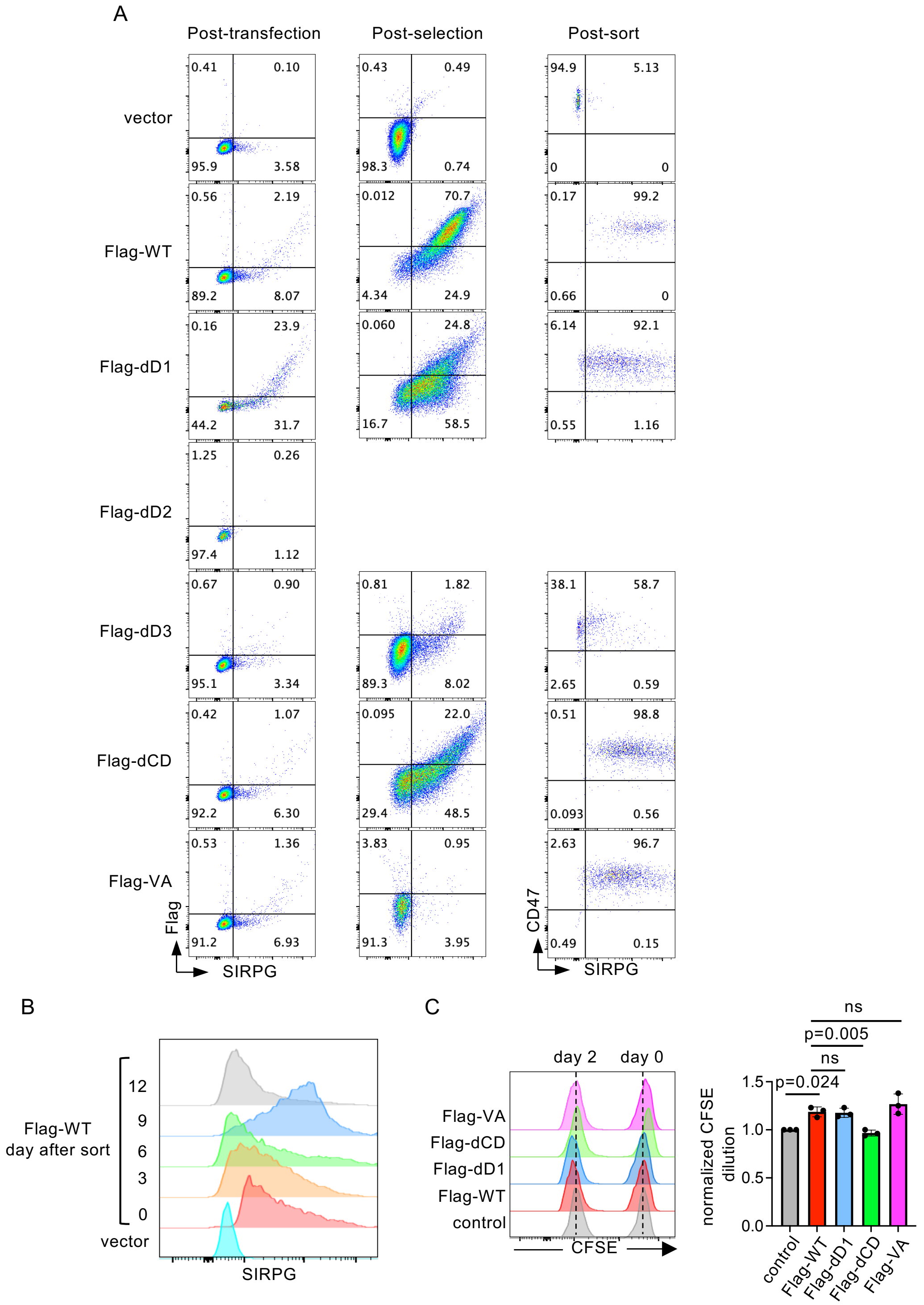


**Figure S5. Structural and functional analyses of SIRPG. A-C.** SIRPG KO Jurkat cells were transfected with indicate Flag-SIRPG constructs shown in Figure 7A. The expression of Flag-SIRPG was examined 3 days after the transfection, 3 weeks after hygromycin selection, and immediately after sorting (**A**). The expression of Flag-WT at different time points after sorting is shown in overlay histograms (**B**). The sorted cells were stained for CFSE and the level of CFSE was examined on day 0 and day 2 (the left panel of **C**). The normalized CFSE dilution over the two-day course from three independent experiments is shown in the right panel of **C**. The fold of CFSE dilution in control cells (ranging from 12 to 36) was arbitrarily set as 1.

Supplementary Table 1: Differentially expressed genes between SIRPG knockout (KO) and wild-type (WT) CD4$^+$ T cells.

| symbol | baseMean | log2FoldChange | lfcSE | stat | pvalue | padj |
|---|---|---|---|---|---|---|
| HLA-DRB5 | 59.78411 | 0.966898 | 0.238051 | 4.061726 | 4.87E-05 | 0.007745 |
| TC2N | 93.46348 | 0.650992 | 0.174647 | 3.727482 | 0.000193 | 0.021707 |
| SYTL2 | 73.50878 | 0.676196 | 0.186249 | 3.630602 | 0.000283 | 0.029163 |
| HLA-DPB1 | 203.5102 | 0.766374 | 0.216427 | 3.541022 | 0.000399 | 0.037097 |
| OASL | 91.39674 | 0.625633 | 0.182334 | 3.431247 | 0.000601 | 0.04679 |
| SIRPG | 140.3094 | -1.7283 | 0.171662 | -10.0681 | 7.65E-24 | 2.92E-20 |
| CCL22 | 98.55052 | -1.32521 | 0.192077 | -6.89938 | 5.22E-12 | 6.64E-09 |
| TOP2A | 282.0382 | -0.67017 | 0.102253 | -6.554 | 5.6E-11 | 4.28E-08 |
| EGR2 | 215.3408 | -0.63162 | 0.110617 | -5.71003 | 1.13E-08 | 7.18E-06 |
| ZBED2 | 241.6874 | -0.62887 | 0.115326 | -5.45297 | 4.95E-08 | 2.7E-05 |
| CDK1 | 104.1192 | -0.88308 | 0.167041 | -5.28662 | 1.25E-07 | 5.94E-05 |
| SEMA4A | 130.0913 | -0.69335 | 0.139731 | -4.96199 | 6.98E-07 | 0.000266 |
| CCL1 | 260.3065 | -0.84869 | 0.188032 | -4.51357 | 6.37E-06 | 0.002211 |
| CCNB1 | 96.33368 | -0.75533 | 0.175337 | -4.30789 | 1.65E-05 | 0.004193 |
| UBE2C | 77.83421 | -0.79782 | 0.186231 | -4.28402 | 1.84E-05 | 0.004378 |
| CD200 | 59.46942 | -0.83705 | 0.205598 | -4.0713 | 4.68E-05 | 0.007745 |
| CRTAM | 94.73216 | -0.83434 | 0.205193 | -4.06611 | 4.78E-05 | 0.007745 |
| TUBA1C | 84.31008 | -0.75188 | 0.187709 | -4.00554 | 6.19E-05 | 0.00939 |
| TPX2 | 106.2511 | -0.59807 | 0.158616 | -3.77053 | 0.000163 | 0.019426 |

Supplementary Table 2: Differentially expressed genes between SIRPG knockout (KO) and wild-type (WT) CD8+ T cells.

| symbol | baseMean | log2FoldChange | lfcSE | stat | pvalue | padj |
|---|---|---|---|---|---|---|
| GIMAP4 | 215.5172 | 0.594756 | 0.148682 | 4.000184 | 6.33E-05 | 0.012228 |
| XAF1 | 134.1174 | 0.693915 | 0.189259 | 3.666492 | 0.000246 | 0.037116 |
| TXNIP | 130.8879 | 0.632712 | 0.178213 | 3.550316 | 0.000385 | 0.047361 |
| XCL2 | 446.339 | -1.10529 | 0.142245 | -7.77033 | 7.83E-15 | 3.78E-11 |
| XCL1 | 506.1353 | -1.00745 | 0.135113 | -7.45635 | 8.9E-14 | 2.15E-10 |
| SIRPG | 144.3338 | -1.48033 | 0.203503 | -7.27425 | 3.48E-13 | 4.96E-10 |
| CCL1 | 210.1461 | -1.29386 | 0.178413 | -7.25206 | 4.1E-13 | 4.96E-10 |
| CRTAM | 413.4742 | -0.7874 | 0.11496 | -6.84932 | 7.42E-12 | 7.17E-09 |
| TOP2A | 395.6591 | -0.7975 | 0.132677 | -6.01087 | 1.85E-09 | 1.49E-06 |
| KPNA2 | 333.735 | -0.72861 | 0.129715 | -5.61704 | 1.94E-08 | 1.34E-05 |
| UBE2C | 103.0297 | -0.97202 | 0.203792 | -4.76968 | 1.85E-06 | 0.000891 |
| EGR2 | 271.7175 | -0.5911 | 0.132796 | -4.45123 | 8.54E-06 | 0.002946 |
| TUBB4B | 229.0424 | -0.6924 | 0.156542 | -4.42313 | 9.73E-06 | 0.003132 |
| CDK1 | 154.6944 | -0.87191 | 0.198119 | -4.40096 | 1.08E-05 | 0.003253 |
| RRM2 | 468.2425 | -0.58502 | 0.134024 | -4.36502 | 1.27E-05 | 0.003611 |
| TUBA1C | 87.56303 | -0.99333 | 0.228813 | -4.34121 | 1.42E-05 | 0.003802 |
| AURKA | 73.70983 | -1.05291 | 0.24539 | -4.29076 | 1.78E-05 | 0.004526 |
| TNFRSF18 | 96.11126 | -0.90015 | 0.212658 | -4.23286 | 2.31E-05 | 0.005572 |
| CCNB1 | 128.1495 | -0.84301 | 0.205557 | -4.10108 | 4.11E-05 | 0.009458 |
| CDC20 | 155.1565 | -0.78789 | 0.195631 | -4.02743 | 5.64E-05 | 0.011348 |
| RGS16 | 71.68637 | -1.03311 | 0.255506 | -4.0434 | 5.27E-05 | 0.011348 |
| PDLIM4 | 76.23234 | -1.01915 | 0.257739 | -3.95418 | 7.68E-05 | 0.014267 |
| TNFRSF9 | 280.3794 | -0.58078 | 0.152881 | -3.79891 | 0.000145 | 0.025701 |
| TPX2 | 139.1278 | -0.71002 | 0.193029 | -3.6783 | 0.000235 | 0.037116 |
| CCL22 | 140.6534 | -2.19652 | 0.59861 | -3.66936 | 0.000243 | 0.037116 |
| CD83 | 44.60081 | -1.15075 | 0.312864 | -3.67812 | 0.000235 | 0.037116 |
| RACGAP1 | 66.79175 | -1.00827 | 0.278598 | -3.61909 | 0.000296 | 0.039665 |
| FABP5 | 184.4237 | -0.68046 | 0.187926 | -3.62088 | 0.000294 | 0.039665 |
| AURKB | 79.64339 | -0.82549 | 0.227499 | -3.62854 | 0.000285 | 0.039665 |
| APOBEC3B | 89.25181 | -1.01529 | 0.27916 | -3.63695 | 0.000276 | 0.039665 |
| TUBA1B | 1758.407 | -0.61996 | 0.173194 | -3.57957 | 0.000344 | 0.043744 |
| CD72 | 43.39631 | -1.05958 | 0.299962 | -3.53238 | 0.000412 | 0.047361 |

Supplementary Table 3: Differentially expressed genes between SIRPG knockout (KO) and wild-type (WT) including both CD4+ and CD8+ T cells.

| symbol | baseMean | log2FoldChange | lfcSE | stat | pvalue | padj |
|---|---|---|---|---|---|---|
| HLA-DPB1 | 207.0103 | 0.641926 | 0.11056 | 5.806144 | 6.39E-09 | 1.44E-06 |
| SYTL2 | 83.20699 | 0.61851 | 0.122724 | 5.039855 | 4.66E-07 | 6.64E-05 |
| TC2N | 79.07288 | 0.65012 | 0.133006 | 4.887905 | 1.02E-06 | 0.000129 |
| OASL | 91.97102 | 0.601569 | 0.132592 | 4.537002 | 5.71E-06 | 0.00049 |
| RGS1 | 104.4287 | 0.624296 | 0.141178 | 4.422058 | 9.78E-06 | 0.000772 |
| PLEK | 47.05728 | 1.296842 | 0.311173 | 4.167593 | 3.08E-05 | 0.002044 |
| HLA-DRB5 | 69.53112 | 0.71695 | 0.17816 | 4.024192 | 5.72E-05 | 0.003506 |
| IL7R | 71.10164 | 0.605254 | 0.161117 | 3.756607 | 0.000172 | 0.007889 |
| TRG-AS1 | 41.6858 | 0.616783 | 0.169274 | 3.643698 | 0.000269 | 0.011139 |
| PDE4B | 38.80476 | 0.604913 | 0.173555 | 3.485429 | 0.000491 | 0.017725 |
| TRGC2 | 38.93226 | 0.730415 | 0.218617 | 3.34107 | 0.000835 | 0.025805 |
| HLA-DRB1 | 207.4469 | 0.632239 | 0.195641 | 3.231633 | 0.001231 | 0.03377 |
| NBPF26 | 53.13375 | 0.996015 | 0.317954 | 3.132579 | 0.001733 | 0.043837 |
| SIRPG | 141.7013 | -1.63075 | 0.132064 | -12.3482 | 4.98E-35 | 2.91E-31 |
| TOP2A | 325.722 | -0.72594 | 0.076646 | -9.47139 | 2.76E-21 | 8.07E-18 |
| EGR2 | 236.8204 | -0.61897 | 0.073522 | -8.4188 | 3.8E-17 | 5.56E-14 |
| CRTAM | 218.7413 | -0.81642 | 0.101878 | -8.01372 | 1.11E-15 | 1.3E-12 |
| ZBED2 | 314.0547 | -0.58544 | 0.073323 | -7.98439 | 1.41E-15 | 1.38E-12 |
| KPNA2 | 266.783 | -0.60033 | 0.07901 | -7.5981 | 3.01E-14 | 2.51E-11 |
| CDK1 | 123.5665 | -0.8777 | 0.121229 | -7.24006 | 4.49E-13 | 3.28E-10 |
| UBE2C | 87.48298 | -0.86732 | 0.124271 | -6.97922 | 2.97E-12 | 1.93E-09 |
| CCL1 | 240.1543 | -1.05099 | 0.154247 | -6.8137 | 9.51E-12 | 5.56E-09 |
| CCL22 | 114.7806 | -1.66006 | 0.264603 | -6.27377 | 3.52E-10 | 1.29E-07 |
| CCNB1 | 108.4851 | -0.79172 | 0.126105 | -6.27827 | 3.42E-10 | 1.29E-07 |
| XCL2 | 190.6097 | -1.27163 | 0.204212 | -6.227 | 4.75E-10 | 1.63E-07 |
| TUBA1C | 85.37743 | -0.84997 | 0.137155 | -6.19718 | 5.75E-10 | 1.77E-07 |
| CD200 | 62.7888 | -0.81032 | 0.139997 | -5.7881 | 7.12E-09 | 1.54E-06 |
| TPX2 | 118.7997 | -0.64339 | 0.112584 | -5.71477 | 1.1E-08 | 2.29E-06 |
| TNFRSF18 | 156.0165 | -0.61551 | 0.111756 | -5.50761 | 3.64E-08 | 7.33E-06 |
| AURKB | 67.30321 | -0.68557 | 0.13456 | -5.09492 | 3.49E-07 | 5.1E-05 |
| FABP5 | 113.9944 | -0.64324 | 0.130358 | -4.9344 | 8.04E-07 | 0.000107 |
| PLK1 | 94.39407 | -0.60193 | 0.123293 | -4.88212 | 1.05E-06 | 0.00013 |
| AURKA | 63.49402 | -0.7965 | 0.163934 | -4.85868 | 1.18E-06 | 0.000144 |
| XCL1 | 230.5397 | -0.87918 | 0.181734 | -4.83772 | 1.31E-06 | 0.000157 |
| CDC20 | 119.4076 | -0.58653 | 0.124917 | -4.69533 | 2.66E-06 | 0.000259 |
| RGS16 | 45.42847 | -0.80737 | 0.172204 | -4.68846 | 2.75E-06 | 0.000264 |
| HJURP | 49.45906 | -0.74368 | 0.15953 | -4.66169 | 3.14E-06 | 0.000296 |
| CCNA2 | 101.3999 | -0.60692 | 0.133681 | -4.5401 | 5.62E-06 | 0.00049 |
| SPAG5 | 74.54383 | -0.5929 | 0.135065 | -4.38979 | 1.13E-05 | 0.000881 |
| CCNB2 | 90.07022 | -0.60526 | 0.137944 | -4.38769 | 1.15E-05 | 0.000881 |
| PDLIM4 | 52.97124 | -0.79408 | 0.181356 | -4.37857 | 1.19E-05 | 0.000907 |

| | | | | | | |
|---|---|---|---|---|---|---|
| KIF23 | 60.43094 | -0.62582 | 0.143994 | -4.34614 | 1.39E-05 | 0.001038 |
| RACGAP1 | 57.86942 | -0.71329 | 0.164652 | -4.33213 | 1.48E-05 | 0.001092 |
| CDCA5 | 70.74992 | -0.593 | 0.141329 | -4.1959 | 2.72E-05 | 0.001891 |
| APOBEC3B | 60.80502 | -0.764 | 0.183197 | -4.17038 | 3.04E-05 | 0.002043 |
| KIFC1 | 30.41791 | -0.81942 | 0.203723 | -4.02224 | 5.76E-05 | 0.003506 |
| KIF20A | 47.40967 | -0.66754 | 0.167585 | -3.98331 | 6.8E-05 | 0.003894 |
| IL18R1 | 38.14404 | -0.71126 | 0.180252 | -3.94594 | 7.95E-05 | 0.004424 |
| CAV1 | 49.46489 | -0.65875 | 0.171743 | -3.83564 | 0.000125 | 0.006364 |
| IL13 | 45.44287 | -0.77858 | 0.203576 | -3.8245 | 0.000131 | 0.00649 |
| CD83 | 54.45562 | -0.63803 | 0.168937 | -3.77674 | 0.000159 | 0.007549 |
| CDC45 | 57.58178 | -0.59679 | 0.164809 | -3.62109 | 0.000293 | 0.011989 |
| HMMR | 39.17415 | -0.624 | 0.173949 | -3.58728 | 0.000334 | 0.013503 |
| LINC01281 | 42.42188 | -0.59939 | 0.168103 | -3.56559 | 0.000363 | 0.014335 |
| EXO1 | 33.29814 | -0.69232 | 0.196385 | -3.52532 | 0.000423 | 0.015948 |
| TYMS | 171.4017 | -0.68717 | 0.200636 | -3.42497 | 0.000615 | 0.020651 |
| CDCA2 | 31.56426 | -0.67213 | 0.197156 | -3.40911 | 0.000652 | 0.021519 |
| NR4A2 | 31.7803 | -0.7202 | 0.212016 | -3.39692 | 0.000681 | 0.022375 |
| TROAP | 34.27303 | -0.6042 | 0.181153 | -3.33533 | 0.000852 | 0.026205 |
| LMNB2 | 34.4321 | -0.6087 | 0.187651 | -3.2438 | 0.001179 | 0.033139 |

Supplemental Table 4: Antibody resource table

| Reagent Type | Designation | Source | Category Number | Host and subclass | Clone name |
|---|---|---|---|---|---|
| Antibody | Anti-human CD3 | BioLegend | 317330 | Mouse IgG2a, k | OKT3 |
| Antibody | Purified anti-human CD3 | BioLegend | 317302 | Mouse IgG2a, k | OKT3 |
| Antibody | Anti-human CD4 | BioLegend | 344622 | Mouse IgG1, k | SK3 |
| Antibody | Anti-human CD4 | BioLegend | 317450 | Mouse IgG2a, k | OKT4 |
| Antibody | Anti-human CD8 | BioLegend | 344732 | Mouse IgG1, k | SK1 |
| Antibody | Anti-human CD8 | BioLegend | 344712 | Mouse IgG1, k | SK1 |
| Antibody | Anti-human SIRPG | BioLegend | 336606 | Mouse IgG1, k | LSB2.20 |
| Antibody | Anti-human CD45RO | BioLegend | 304222 | Mouse IgG2a, k | UCHL1 |
| Antibody | Anti-human CD45RA | BioLegend | 304120 | Mouse IgG2a, k | HI100 |
| Antibody | Anti-human CD27 | BioLegend | 986910 | Mouse IgG1, k | M-T271 |
| Antibody | Anti-human CD28 | BioLegend | 302928 | Mouse IgG1, k | CD28.2 |
| Antibody | Ultra-LEAF™ Purified anti-human CD28 Antibody | BioLegend | 302934 | Mouse IgG1, k | CD28.2 |
| Antibody | Anti-human CD62L | BioLegend | 304809 | Mouse IgG1, k | DREG-56 |
| Antibody | Anti-human CCR7 | BioLegend | 353223 | Mouse IgG2a, k | G043H7 |
| Antibody | Anti-human GZMK | BioLegend | 370508 | Mouse IgG1, k | GM26E7 |
| Antibody | Anti-human GZMB | BioLegend | 515406 | Mouse IgG1, k | GB11 |

Supplemental Table 5: qPCR primers resource table

| Gene name | Forward primer | Reverse primer |
|---|---|---|
| UBE2C | GATGACCCTCATGGCAGTGG | TTCTCTGGGACCGGACAGTA |
| TOP2A | GGGGTCCTGCCTGTTTAGTC | AGGCTGCAATGGTGACACTT |
| CCNB1 | CCCCTGCAGAAGAAGACCTG | AGTGACTTCCCGACCCAGTA |
| CRTAM | TGTGCCTAACGTAACCCTGC | TGAAAGGAGTTGCCAGCACA |
| TNFRSF18 | CACCCAGTTCGGGTTTCTCA | ACATGCACTGACTCCTCAGC |
| XCL1 | TCTGGCTAGTGTCTATCAGAGGT | ATGGGAACCCAGTGAAGACT |
| GAPDH | GACAGTCAGCCGCATCTTCT | GCGCCCAATACGACCAAATC |
